# Analysis of the Bin1 SH3 interaction with peptides derived from the hepatitis C virus protein NS5A and c-Myc reveals that NS5A can competitively displace c-Myc *in vitro*

**DOI:** 10.1186/2047-783X-19-S1-S10

**Published:** 2014-06-19

**Authors:** Amine Aladağ, Christina Bösing, Lothar Gremer, Silke Hoffmann, Stefan Klinker, Melanie Schwarten, Matthias Stoldt, Olga Valdau, Dieter Willbold

**Affiliations:** 1 Institute of Physical Biology, Heinrich Heine University, 40225 Düsseldorf, Germany; 2 Institute of Complex Systems (ICS-6) Structural Biochemistry, Forschungszentrum Jülich, 52425 Jülich, Germany

## Background

Severe liver damage like cirrhosis and hepatocellular carcinoma (HCC) can be caused by manifestation of the hepatitis C virus (HCV) infection. Constitutively activated c-Myc oncogene has been shown to contribute to the establishment of HCV-mediated HCC. Interestingly, only one of many isoforms of the tumor suppressor protein Bin1 (bridging integrator 1), Bin1+12A, contains an internal, canonical SH3 binding motif that recognizes its own SH3 domain. This leads to the inability of Bin1+12A to interact with c-Myc. The expression of the Bin1+12A isoform is a main phenotype in malignant melanoma cells. We suggest that also other mechanisms that disturb the interaction of Bin1 and c-Myc might have severe consequences since the latter is tightly regulated in healthy cells. The HCV nonstructural protein 5A (NS5A) plays a key role in virus replication and assembly. NS5A plays an intercepting role in several cellular pathways, which are linked to cell growth, cell cycle control, cell survival, cellular stress response, apoptosis as well as HCC. It is known that NS5A contains a highly conserved canonical, poly-proline (PxxP) SH3-binding motif, which is located between its D2 and D3 domains. This PxxP motif was described to interact with the SH3 domain of Bin1. In addition to a biophysical analysis of the canonical binding between Bin1 SH3 and the PxxP motif of NS5A [1], we identified two additional low-affinity binding sites for noncanonical SH3 binding on NS5A [2]. The hypothesis underlying the work presented here is that viral NS5A is able to sequester cellular Bin1 from c-Myc.

## Materials and methods

Expression and purification of Bin1 SH3 was done according to published procedures [3]. NS5A(347-361) and c-Myc(55-69) peptides were from GeneScript (USA) as reversed-phase HPLC purified and mass spectroscopy confirmed products. Fluorescence titration measurements were done, and data were fitted as previously described [3]. To obtain titration curves for deriving the binding constants, peptides from stock solutions in the same buffer were added in small increments to free SH3 domain (0.5 μM, 1x PBS with 2 mM DTT). Nuclear magnetic resonance (NMR) spectroscopy was performed using a Varian UnityINOVA NMR spectrometer (Varian, Palo Alto, USA, 600 MHz proton frequency, cryogenically cooled 5 mm Z-PFG-1H (^13^C, ^15^N)-probe head, 298 K). The peptides c Myc(55-69) and NS5A(347-361) were added stepwise to free SH3 domain (0.1 mM [*U*-^15^N]-Bin1 SH3, 1xPBS, 2 mM DTT, 10% (v/v) deuterium oxide) until 1:1 ratios were reached. Competition experiments were conducted by adding c-Myc(55-69) peptide to a preformed Bin1 SH3:NS5A(347-361) complex and by adding NS5A(347-361) peptide to a preformed Bin1 SH3:c-Myc(55-69) complex until final ratios of 1:1:1 have been reached. Spectra were processed (NMRPipe) and analyzed (CcpNMR). New resonance signals of the Bin1 SH3:NS5A(347-361) complex were assigned using a 3D HNCACB of [*U*-^13^C, U-^15^N]-Bin1 SH3 in complex with an NS5A(333-369) peptide. The weighted chemical shift changes upon interaction were calculated as described [3].

## Results

Using fluorescence titration we calculated the dissociation constant (K*_D_*) as 0.2 ± 0.1 μM for the interaction between Bin1 SH3 and NS5A(347-361), comprising the core SH3 binding motif of NS5A. This value is consistent with that for a longer NS5A(333-369) peptide determined previously by surface plasmon resonance [1], and is approximately 40-fold higher than the published value of 4.2 µM for the affinity between Bin1 SH3 and a 15-mer c-Myc peptide (residues 55 to 69) comprising the c-Myc SH3 binding side [4]. Our results suggest that an NS5A derived peptide should be able to easily compete with the c-Myc peptide for binding to Bin1 SH3.

Since the interaction surface of a protein as well as the strength of an interaction can be defined by the observation of its chemical shift perturbation (CSP) upon ligand binding, we measured (^1^H-^15^N)-HSQC spectra of [*U*-^15^N]-Bin1 SH3 either in the absence or in the presence of increasing c Myc(55-69) concentrations. The spectra overlay clearly shows a gradual chemical shift change indicating a fast exchange regime of free and bound protein typical for low affinity binding that is common for most SH3:ligand interactions. In contrast, and consistent with the low K*_D_* measured by tryptophan fluorescence quenching, adding of NS5A(347-361) to [*U*-^15^N]-Bin1 SH3 resulted in decreasing intensities of the signals correlated with the free-state of Bin1 SH3 accompanied with the appearance of new resonance signals, which correspond to the Bin1 SH3:NS5A(347-361) complex. This behavior is known to be typical for slow complex dissociation observed for strong interactions between a protein and a ligand. The different exchange kinetics observed for the Bin1 SH3:NS5A(347-361) complex and for the Bin1 SH3:c-Myc(55-69) complex indicate a significantly higher affinity of the Bin1 SH3 domain to the peptide derived from NS5A than to that one derived from c-Myc. To probe the competing potential of NS5A, we performed an additional (^1^H-^15^N)-HSQC experiment and added NS5A(347-361) to an existing Bin1 SH3:c-Myc(55-69) complex in solution. Comparison of this spectrum with the spectrum of the Bin1 SH3:NS5A(347-361) complex revealed that both spectra are virtually identical, a fact, which can be explained by a NS5A(347-361) mediated displacement of c-Myc(55-69) from the Bin1 SH3 binding surface.

Finally, we combined our CSP data with own unpublished assignment data and those of PDB entry for free Bin1 SH3 (PDB: 1MUZ) to visualize the binding interfaces of NS5A(347-361) and c-Myc(55-69) with Bin1 SH3. As expected both peptides follow the canonical PxxP-ligand binding mode, and interact via Bin1 SH3 residues in the RT loop, the C-terminal region of the n-Src loop and β-strands 4 and 5. Bin1 SH3 domain is one of most negatively charged SH3 domains, and several of these residues are located in the Bin1 SH3 binding interface. Thereby, the negatively charged aspartate residues 535 and 537 in the RT-loop, which are unique for the Bin1 SH3 domain, seem to be the main determinants for the NS5A:Bin1 SH3 interaction. This probably explains why ligands with many positive residues such as NS5A can be bound by Bin1 SH3 with the observed extraordinary high specificity and affinity.

## Conclusions

Typically, the interaction between a cellular SH3 domain and a cellular ligand is transient and the respective affinity is in the high to low micromolar range. Hence, SH3 domains usually are optimized to be able to choose between many cellular partners of diverse cellular pathways in respect to the cellular needs and environmental factors. Our data show that a short peptide comprising the canonical PxxP binding motif located inside the low complexity sequence 2 region of NS5A is able to easily compete with a c-Myc-derived peptide resembling the physiologic Bin1 SH3 binding site of c-Myc *in vitro*. This provides first insights into not yet recognized activities of the HCV NS5A protein. We suggest that NS5A might have the potency to negatively influence the SH3 mediated tumor suppressor qualities of Bin1 also *in vivo*, e.g. by enhancing the levels of free and activated c-Myc molecules inside HCV infected cells.
